# Molecular Determinants Regulating the Plasticity of the MHC Class II Immunopeptidome

**DOI:** 10.3389/fimmu.2022.878271

**Published:** 2022-05-16

**Authors:** Laura Santambrogio

**Affiliations:** ^1^Caryl and Israel Englander Institute for Precision Medicine, Weill Cornell Medicine, New York, NY, United States; ^2^Department of Radiation Oncology, Weill Cornell Medicine, New York, NY, United States; ^3^Sandra and Edward Meyer Cancer Center, Weill Cornell Medicine, New York, NY, United States

**Keywords:** MHC class II, antigen processing and presentation, dendritic cells, HLA-DM, HLA-DO

## Abstract

In the last few years, advancement in the analysis of the MHC class II (MHC-II) ligandome in several mouse and human haplotypes has increased our understanding of the molecular components that regulate the range and selection of the MHC-II presented peptides, from MHC class II molecule polymorphisms to the recognition of different conformers, functional differences in endosomal processing along the endocytic tract, and the interplay between the MHC class II chaperones DM and DO. The sum of all these variables contributes, qualitatively and quantitatively, to the composition of the MHC II ligandome, altogether ensuring that the immunopeptidome landscape is highly sensitive to any changes in the composition of the intra- and extracellular proteome for a comprehensive survey of the microenvironment for MHC II presentation to CD4 T cells.

## MHC Class II Polymorphism

The gene clusters encoding MHC-II proteins are among the most polymorphic genes in the genome. They are closely linked in all species (on chromosome 2 in humans and chromosome 17 in mice) and therefore inherited together. Most of our knowledge of the immunological relevance of MHC-II proteins derives from the analysis of patients with MHC II deficiency due to a failure in MHC-II transcription (1). Lack of MHC-II expression results in severe compromise of CD4+ T cell development and a consequent fatal immunodeficiency (bare lymphocyte syndrome) (1). Similarly, mice lacking MHC-II experience a near-complete elimination of CD4+ T cells from secondary lymphatic organs, and the thymus is populated by immature CD4+ thymocytes (2). Even a less severe MHC-II deficiency (eightfold reduction in MHC class II expression) leads to a suboptimal T cell response, increased autoimmunity, and altered cytokine inducibility (3).

The major “classical” MHC-II proteins are designated as HLA-DR (DR), HLA-DP (DP), and HLA-DQ (DQ) in humans and H2-A and E in mice. Each of the HLA and H2 loci contains genes coding for the alpha (DPA, DRA, or DQA in humans and A-alpha E-alpha in mice) and beta chain (DPB, DRB, or DQB in humans and A-beta E-beta in mice) proteins (4–6). Additionally, each of the classical MHC-II genes contains several alternate alleles; the combination of alleles on a given chromosome is called a haplotype, and currently, human alleles/haplotypes have been mapped in the thousands (http://hla.alleles.org/nomenclature/index.html), underscoring the diversity of MHC-II proteins across individuals and establishing the molecular basis for the differences in immune response across the population. Both MHC alleles in any one individual are co-dominantly expressed without allelic exclusion and inherited in a Mendelian fashion from each parent; as such, each individual, will express both maternally and paternally derived haplotypes.

Each MHC class II molecule assembles as a dimeric protein pairing one alpha and one beta chain, which form an open-ended groove capable of binding peptides of 13–25 amino acids in length (7). Professional antigen presenting cells (APCs; dendritic cells, macrophages) will express two unique DP, DQ, and DR isoforms (IA and IE in mice), albeit some individuals express additional DR alleles. The MHC-II polymorphism is mostly clustered in the peptide-binding cleft (5, 6), where conserved amino acid residues located at key positions of the peptides act as anchoring residues in the MHC-II-binding groove determining the peptide-binding specificity of each MHC-II haplotype (8).

Besides affecting peptide selection/binding, the MHC-II polymorphism also affects interaction with its chaperone invariant chain (Ii). Invariant Chain, assemble in the endoplasmic reticulum (ER), with the MHC II α and β chains to prevent binding of ER-derived peptides and, through its Leucine/Isoleucin endosomal targeting signals, facilitate MHC II endosomal traffic, *via* de plasma membrane (9–11). The Invariant Chain, prevents ER-peptides binding by inserting one of a nested set of peptides, known as CLIPs (class II-associated Ii peptides), into the MHC II binding groove (10). The presence of at least three CLIP peptides, comprising the Ii sequence between aa 87 and 107, and their ability to bind in different registers, allows the promiscuity of CLIPs binding to various MHC II molecules.

Analysis of MHC-II trafficking in mice has shown that in the absence of Ii, the I-Ab haplotype aggregates in the ER, and surface MHC-II complexes have an unstable “floppy” MHC II conformation. On the other hand, the I-Ak and I-Ad haplotypes assemble more efficiently and are more conformationally similar to mature wild-type MHC (12–14). Additionally, although Ii-lacking cells are generally defective in presenting intact antigens, all haplotypes can efficiently load peptides at the cell surface (15).

In humans, analysis of different haplotypes has also shown heterogenicity in response to the absence of Ii. From the DR1 (DRA, DRB1*0101) haplotype which is highly dependent on Ii for MHC II trafficking and assembly, to the HLA-DP alleles carrying the 84Gly beta chain polymorphism, which fail to bind CLIP, and can assemble as empty molecules, and still bind exogenously derived peptides at the cell surface (16). Analysis of CLIP stability also indicated that some haplotypes (DR*0401 and DR*0404) are less stable than others (DR*0402), and the haplotypes with reduced CLIP stability are more prone to autoimmunity (17).

Quantitative differences in expression and translation of MHC-II proteins is also accountable for the unequal expression among the different alleles, with DR in humans and I-A in mice being the most abundant as compared with DQ, DP, and IE, respectively (18).

Additionally, CIITA, the master regulator of constitutive and inducible MHC-II expression, is controlled by different promoters in different professional and nonprofessional APCs, with promoter I being the most represented in DCs and macrophages, promoter III constitutively expressed in B cells and pDCs, and promoter IV being induced by IFN-γ in both hematopoietic and non-hematopoietic cells (19–21). Additionally, CIITA variants with reduced transactivation function have been reported, altogether further expanding the quantitative differences in MHC-II expression among APCs.

Finally, MHC-II molecules can also exist in two distinct conformational states due to duplicate GXXXG motifs in the alpha chain transmembrane region, which enable alternative pairing with the single motif in the beta chain, generating one of two possible conformations (M1 or M2). M1 is the less represented conformation; however, it is uniquely linked to B cell activation and presentation of peptides derived from the processing of B cell receptor (BCR)-bound cognate antigens. On the other hand, antigens phagocytosed by fluid phase endocytosis are loaded on both M1 and M2 conformations (22, 23).

Finally, a previously unidentified variant of mouse I-A^s^ (Pro vs Ala) at residue 58 induces changes to MHC-II structure and conformational stability, skewing peptide binding/recognition *via* alternate docking modes (24).

In sum, the level of MHC-II expression on professional and nonprofessional APCs, as controlled by CIITA variants and the pro-inflammatory environment; unequal expression of different alleles; M1 and M2 conformations; and, most importantly, the MHC- II intrinsic polymorphism all contribute to the quantitative and qualitative differences in MHC-II -restricted presentation.

## Sources of MHC II Peptides

**Figure 1:**
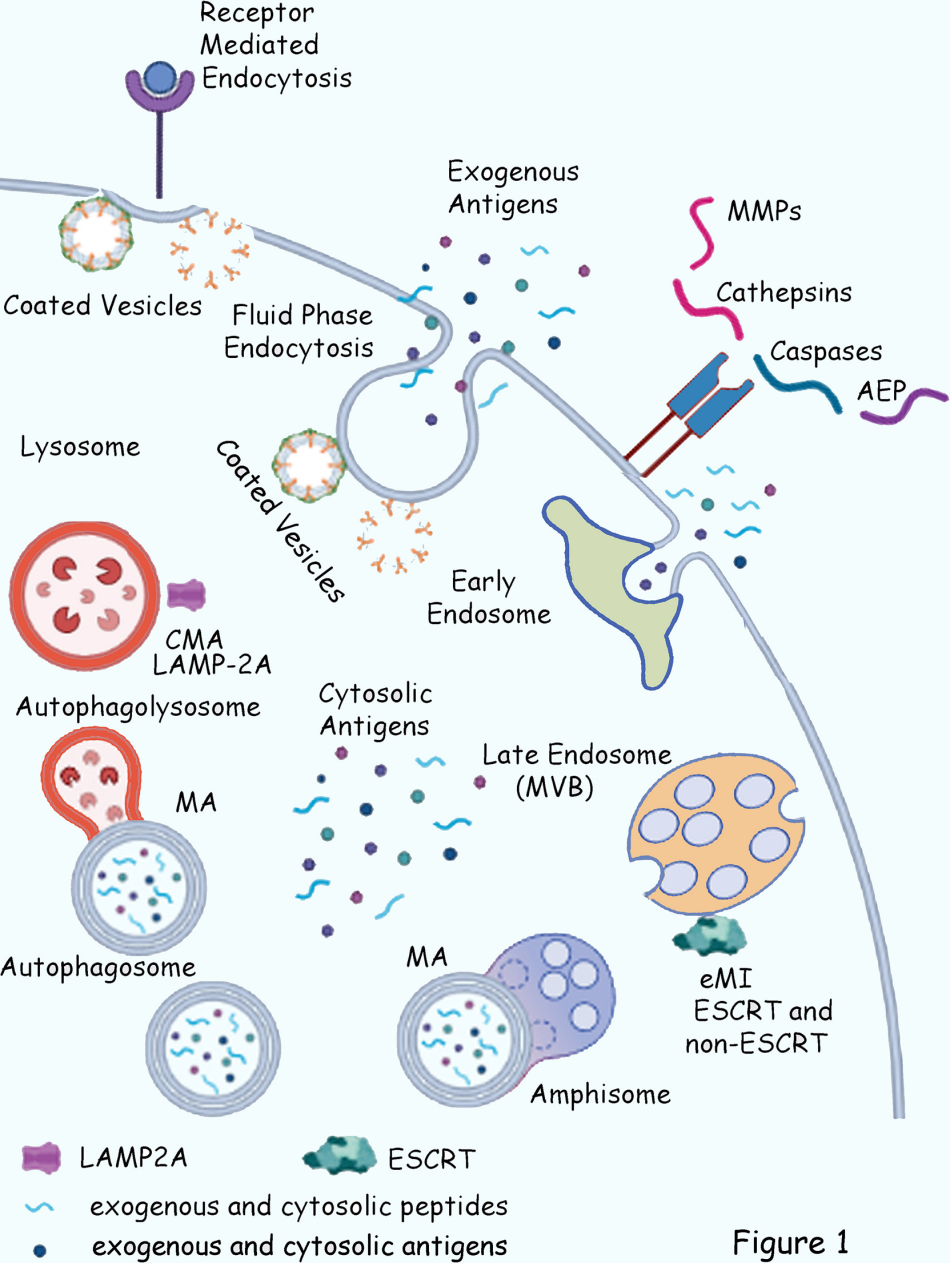
Schematic of the cytosolic and exogenous pathways for antigen delivery to MHC II compartments. Exogenous antigens are acquired through fluid phase endocytosis and receptor-mediated endocytosis. Cytosolic proteins enter MHC II compartments through Macroautophagy (MA), upon fusion of the Autophagosomes with Lysosomes (Autophagolysosome) or with Late Endosomes (Amphisome); Endosomal Microautophagy (eMI, ESCRT-dependent and independent), and LAMP2A-mediated chaperone-mediated autophagy (CMA). Additional antigen-acquisition routes include preprocessed cytosolic proteasome-generated peptides, as well as acquisition of extracellular peptides present in biological fluids loaded on plasma membrane and early-endosomes recycling MHC-II molecules. As such, MHC-II-eluted self-peptides derived from a variety of processing pathways including Cathepsins, Caspases and other endopeptidase such as asparagine endopeptidase (AEP), and matrix metalloproteases (MMPs), among many others.

There are two main sources of proteins entering the MHC-II compartments: extracellular and cytosolic (25–27) (**Figure 1**). Exogenous proteins are continuously acquired through phagocytosis, macro/micropinocytosis, and receptor-mediated endocytosis, particularly in APCs with high phagocytic activity, such as DCs and macrophages (27). B cells mostly rely on BCR-mediated endocytosis (28), although they can internalize extracellular antigens by fluid phase endocytosis when present at high concentrations (29).

Cytosolic proteins enter endosomal compartments through different forms of autophagy, macroautophagy (MA), endosomal microautophagy (eMI), and chaperone-mediated autophagy (CMA) (25, 30, 31) (**Figure 1**). Additional antigen-acquisition routes include preprocessed cytosolic proteasome-generated peptides (32, 33), as well as acquisition of extracellular peptides present in biological fluids loaded on plasma membrane and recycling of MHC-II molecules (34–37) (**Figure 1**). Indeed, using experimental protease databases, it was determined that MHC-II-eluted self-peptides presented by immature conventional DCs (cDCs) derived from a variety of processing pathways including cathepsins (cysteine, aspartate and serine proteases), and other endopeptidase such as asparagine endopeptidase (AEP), caspases, complements, and matrix metalloproteases, among many others (38) (**Figure 1**).

Any pathological conditions that change protein synthesis rate or protein afflux to the endosomal compartments will, by default, change the immunopeptidome composition.

For example, in chronic metabolic conditions, such as type II diabetes, the number of MHC-II presented peptides derived from stress-related proteins, metabolic enzymes, and lipoproteins was found to be greatly enhanced (39). Among those, the increase in Apo-B epitope copy number contributed to tilting the immune response from tolerance to immunity with several autoreactive, anti-Apo-B T cells infiltrating the aorta and worsening the inflammatory process (39).The increase in Apo-B MHC-II presentation was not related to MHC-II plasma membrane upregulation because peptides were eluted from the same amounts of MHC-II molecules as quantified by ELISA (39). Similarly, the comparative analysis of the MHC-II immunopeptidome eluted from nodal APCs under physiological or inflammatory conditions clearly identified changes in the immunopeptidome uniquely associated with the inflammatory process, indicating that the increased transcription of adaptive immunity-associated proteins was mirrored in the eluted MHC-II immunopeptidome (40). Additionally, MHC-II elution from both autoimmunity, or *in vivo* infection models has clearly mapped peptides related to the autoimmune process, as well as map pathogen-specific peptides, respectively (41–46). Finally, conditions that increase autophagic flux to the endosomes or increase rate of phagocytosis have been shown to skew the presented immunopeptidome toward the incoming protein source (25, 30, 47, 48).

Altogether, with the advancement in sensitivity and reproducibility of mass spectrometry peptide mapping, it has been possible to quantify changes in MHC-II peptidome, as well as detect peptides represented at low copy number, better than ever before. This technical advancement has allowed for a more comprehensive view of the MHC II ligandome and shown that the immunopeptidome landscape is a balance between intra- and extracellular sources, and it is highly sensitive, and mirrors changes in the cellular proteome. Overall, this ensures that APCs perform a comprehensive survey of the environment for MHC-II presentation to CD4 T cells (38, 49).

## Heterogenicity in the MHC Class II Endosomal Processing Compartments

An additional component that influences the composition of the MHC-II immunopeptidome in professional APCs is the anatomical and molecular organization of the endocytic compartments. Early/recycling endosomes, multivesicular late endosomes, and unilamellar or multilamellar lysosomal compartments are endosomal organelles progressively located along the endocytic tract. The early endosomes, directly derive from the invagination of the plasma membrane, the multivesicular endosomes, are late endosomal compartments, and the lysosomes have been morphologically described as either uni- or multilamellar (50). All these compartments have been shown to be enriched in MHC-II molecules (51). Ultrastructural analysis of these organelles has indicated a variability in their number and morphology among the various types of murine and human APCs and under different cytokine/growth factor conditions. In both human and murine DCs, the major MHC-II compartments have been grouped as either late endosomal multivesicular bodies (MVBs), which may contain few to several internal vesicles, or as multilamellar bodies (MLBs) (50–52), which are formed by onion-like concentric lamellae (50, 52). In immature murine DCs, the most prominent organelle is the multivesicular type (50), whereas in monocyte-derived human DCs, differentiated in granulocyte-macrophage colony-stimulating factor (GM-CSF) and IL-4, both MVBs and MLBs are present, although a bias toward the MLB type has been observed (52). In B cells and macrophages, the MVBs are the most represented MHC-II organelle, and in human monocytes, electrodense unilamellar endosomes and MVBs have been described (53). Additionally, early endosomes, directly deriving from plasma membrane invagination, have also been described as antigen-processing compartments (46, 54–57).

**Figure 2:**
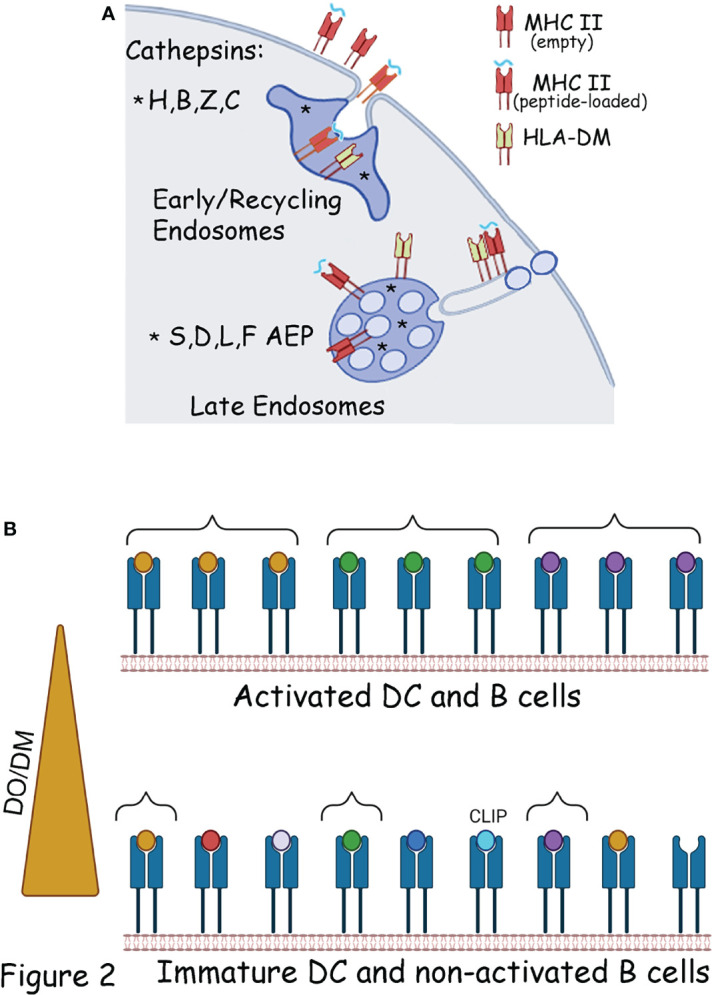
Chaperones and organelles involved in the generation of the MHC II ligandome.**(A)** Endopeptidases (*) present in early and late endosomes, involved in the generation of the MHC II ligandome. In early endosomes the processed peptides will be loaded on MHC II molecule recycling from the plasma membrane. Peptide can also load directly at the cell surface on empty MHC II molecules or in exchange for low affinity peptides. In late endosomes the processing enzymes are located in the lumen of the compartment, outside the vesicles. MHC II molecules are anchored to the vesicles limiting membrane and exposed to the processed peptides. DM is mostly located on the endosomal limiting membrane. As such optimal DM activity is generated during inflammation-mediated endosomal tubulation, which transport MHC II at the cell surface. **(B)** In immature DC, or non-activated B cells, DM activity is inhibited by DO, as such the MHC-II ligandome contains a broad and diverse peptide spectrum, comprises of both low and high affinity peptides and with some empty and MHC-II-CLIP complexes at the cell surface. This MHC-II ligandome facilitate tolerance (Bottom panel) Following DCs and B cell activation the decreased DO activity allows DM to select a more focused, and high affinity MHC-II ligandome to facilitate immunity (Top panel). Braces indicates MHC II molecules in the top and bottom panels, loaded with the same peptide.

All these compartments contain an array of endo/lysosomal hydrolases including proteases, lipases, phosphatases, glycosidases, and nucleases (58). Although the specific and unique contribution of each compartment to antigen processing and MHC-II loading is yet to be fully characterized, important differences are already known. For example, proximal compartments such as early endosomes are enriched in neutral proteases such as cathepsins H, Z and C, (**Figure 2A**) (58, 59) and in these compartments MHC II molecules can exchange Ii for peptide complexes and recycle back the cell surface (60, 61). On the other hand, endosomal and lysosomal compartments are enriched in acid active enzymes such as cathepsins S, B, and D (58, 59, 62, 63) (**Figure 2A**). Other cathepsins, most notably cathepsin L, have been shown to be secreted and function at neutral pH (64).

Additional differences in the protease composition exist among different APCs, with cathepsin F being expressed mostly in macrophages (65) and cathepsins S and L in DCs (63, 66). Exposure to different inflammatory stimuli controls cysteine protease expression at the transcriptional level; for example, pro-inflammatory stimuli have been shown to decrease or increase cathepsin S, L, and B mRNA according to the examined cell type (58). Furthermore, alternative splicing for aspartic and cysteine proteases has also been reported, controlling protein translation, stability, and route of transport, which favor either extracellular secretion or endosomal transport (67). Finally, it has also been reported that cathepsin B, L, and S activities shift from endosomal to lysosomal localization upon IFN-γ treatment (68), likely to avoid extracellular egress of endopeptidase during inflammation-mediated endosomal tubulation for MHC II transport at the cell surface (69). Lastly, developmental regulation of end lysosomal pH has also been reported as a means to control proteolytic activity and peptide generation in DCs (70).

Although a comprehensive analysis of how all these changes in cathepsin translation, location, and activity influence the processing of the MHC-II immunopeptidome has not been conducted yet, it is likely that they have an effect on the generation of the MHC class II ligandome, being the most represented enzyme category in the endo/lysosomal tract.

Indeed, over the years, evidence has indicated that different peptides can be generated in different compartments along the endocytic tract, from the analysis of Ii KO mice, where antigens such as ribonuclease protein was presented equally well in absence of Ii (14), to the processing of myelin basic protein in early endosomes (55, 57) and the fine mapping of different influenza viral peptides being processed either in early or late endosomes (46). Likewise, peptides found in biological fluids, such as lymph, can load on surface MHC-II in immature DCs, as well as on recycling MHC-II molecules in early endosomes (34–36, 38, 71–73). The importance of these compartments, as well as peptides loading at the plasma membrane, has also been emphasized by the intact efficiency of peptides presentation even when MVBs are disrupted (74). On the other hand, globular antigens that need unfolding and S-S bridge reduction require a proteolytic environment as found in late endosomes and lysosomes (49, 75–77).

Altogether, the differential intra and extracellular processing activity (58, 78), generates peptides that can be loaded onto MHC-II proteins at the cell surface (55, 75) or along the endosomal tract, from recycling endosomes (55) to the endo-lysosomes (48, 79). Diversity in antigen location (cytosolic or extracellular), antigen structure (globular or unfolded), organelle processing activity and pH, all contribute to the location along the endocytic tract where peptides are generated.

## Interplay Between HLA-DM and DO in Shaping the MHC II Immunopeptidome

Changes in protein sources (autophagy vs extracellular) and in endosomal processing influence the repertoire of the available peptidome for MHC-II binding. However, among the available peptides, the specificity of the peptide amino acid residues for the polymorphic MHC class II binding groove and the interplay between the MHC-II chaperones HLA-DM and HLA-DO (H2-M and H2-O in mice) will ultimately determine which peptide will occupy the binding groove (80–84). A network of hydrogen bonds and Van der Waals force will then stabilize the non-covalent bond between peptides and MHC-II.

HLA-DM (DM) was discovered in both human and murine cell lines, which were capable of binding exogenous peptides but were much less efficient in presenting internalized proteins, and their surface MHC-II immunopeptidome included several copy numbers of CLIP peptides (81, 85). These results and further studies underscore the enzyme-like activity of DM in exchanging CLIP for antigenic peptides and editing out the low-affinity for high-affinity peptides, altogether ensuring that only stable MHC-II-peptide complexes are transported from the endosomes to the plasma membrane (82, 86). However, DM activity is controlled by the non-polymorphic MHC-II-like molecule HLA-DO (DO). DM and DO associate in the ER and traffic together to the endosomes; more importantly, their interaction is of very high affinity and exceptionally stable, as opposed to the weak DM-MHC class II interaction (87–90). Biochemical studies indicated that DO blocks DM catalyzing activity (90), as later confirmed by crystallographic studies (87). The DO-dependent inhibition of DM activity is developmentally controlled in APCs such as DCs, B cells, and thymic epithelial cells (90). Indeed, DO activity is high in naive B cells and immature DCs, which could explain the presence of CLIP-loaded MHC class II on immature DCs (80), as well as empty MHC-II molecules (34, 35, 52, 91) (**Figure 2B**). On the other hand, upon B and DC maturation, DO is downregulated, as are surface MHC-II-CLIP and empty molecules (80, 92) (**Figure 2B**).

Overall, a broad peptide repertoire, with a limited copy number of each epitope, decreases the possibility of T cell activation. On the other hand, upon B cell and DC activation/maturation, DO downregulation will favor DM catalytic activity and presentation of high-stability peptides is conducive to immunity. Developmentally, this mechanism would favor the MHC-II presentation of a tolerogenic immunopeptidome in immature DC and a more “focused” immunity-inducing peptidome upon DC maturation (**Figure 2B**). This theory has been proven at both the cellular and the MHC-II immunopeptidome level. Early work indicated that HLA-DR1-restricted T cell clones raised against fully competent APCs (DR1+ Ii+ DM+) failed to respond to APCs lacking DM (DR+ Ii- DM- APC) (93, 94). Later on, immunological studies indicated that mice overexpressing DO selected an altered self-peptide repertoire preventing the activation of diabetogenic T cells and subsequent diabetes development (95). At the MHC-II peptidome level, DO-mediated DM inhibition has been suggested to favor immunological tolerance by increasing surface MHC-II-CLIP, as well as to broaden the peptide repertoire by allowing low-stability peptides to be presented. Indeed, characterization of the MHC II peptidome in DO-sufficient and DO-deficient APC, pointed to a set of peptides uniquely present when DO is expressed (96). The same peptides were sensitive to DM-mediated exchange, suggesting that decreased DM editing was responsible for the increased diversity (96). Similarly, analysis of the peptides enriched upon DM expression indicated a higher affinity of the overall peptidome, as compared to the one isolated from low DM expression samples (97).

## Conclusion

Overall, in the last decade, many studies have converged in defining the composition of the MHC-II ligandome at steady state and during pathological conditions. Combined analyses have clearly indicated the plasticity of the MHC-II ligandome, which closely reflects the dynamic changes of the cellular and extracellular proteome composition, the heterogenicity of the processing compartments, and the interplay of the MHC-II molecular chaperones, DO and DM. Altogether, the orchestrated fine-tuning of all the components in the antigen processing and presentation molecular machinery insure the immunosurveillance role of MHC-II molecules.

However, although much progress has been achieved, several open questions remain, for example, how conformational changes in MHC-II structures, related to alpha beta alternative pairing, amino acid variants, or open and empty conformers, contribute to the immune response, or how domains outside the peptide-binding groove drive structural changes and potentially different peptide selection. Additionally, the discovery of the subcellular overlapping between MHC I and MHC II presentation pathways still needs to address how this could change peptide selection. Finally, how the amount of MHC class II epitope copy number influenced by the DO/DM ratio changes the immune response is still open to investigation. Discoveries in these areas will further contribute to our understanding of how MHC II influences both health and disease.

## Author Contributions

The author confirms being the sole contributor of this work and has approved it for publication.

## Funding

This work was supported by the National Institutes of Health (NIH), grant numbers R01AI137198 and R01AI146180.

## Conflict of Interest

The author declares that the research was conducted in the absence of any commercial or financial relationships that could be construed as a potential conflict of interest.

## Publisher’s Note

All claims expressed in this article are solely those of the authors and do not necessarily represent those of their affiliated organizations, or those of the publisher, the editors and the reviewers. Any product that may be evaluated in this article, or claim that may be made by its manufacturer, is not guaranteed or endorsed by the publisher.
